# SfN Journals: Two Paths, One Goal: Sharing Strong Science

**DOI:** 10.1523/ENEURO.0154-16.2016

**Published:** 2016-07-06

**Authors:** Christophe Bernard, Marina Picciotto

Most of our readers know that The Society for Neuroscience now publishes two journals, *The Journal of Neuroscience* and *eNeuro*, but many are puzzled about the difference between them and which journal would be more appropriate for a specific submission. While the journals will be evolving continually, and more differences are likely to appear over time, we would like to start to outline the similarities and differences between the two journals here.

## Structural and Functional

The first difference between the two Society for Neuroscience journals is structural: *eNeuro* is a fully open-access, on-line journal. *eNeuro* publishes articles upon acceptance and does not have formal volumes or issues. *JNeurosci* maintains a print edition that is published weekly, and the on-line issue parallels the print version. Articles in *JNeurosci* are available freely 6 months after publication, and authors can pay an additional fee to make their article open access immediately. The second difference is functional: *eNeuro* can be more experimental in the review process and try new methods to increase the rapidity and transparency of review.

## Peer Review

The system for peer review is different at *eNeuro* and *JNeurosci*, but both journals have a procedure for coming to consensus, particularly when reviewers have disparate views about a manuscript. The review process at *eNeuro* is innovative and involves a double-blind procedure that maintains the anonymity of both the reviewers and the authors. Identifying information is removed before the review process, and authors are asked to eliminate information from the body of the manuscript that identifies the laboratory. After the manuscript is reviewed, reviewers are invited into a consensus review process mediated by the Reviewing Editor to make sure that the decision is reached as a full consensus between reviewers and editor. The Reviewing Editor then provides a review summary laying out exactly what changes or experiments would be needed for acceptance, or why the manuscript was rejected. *The Journal of Neuroscience* uses a tiered review process in which a Reviewing Editor invites the reviewers, evaluates the reviews, and makes a recommendation on whether or not the manuscript is acceptable for publication to one of the Senior Editors. The Senior Editors handle papers from overlapping subsets of the Reviewing Editors, providing a level of consistency for decisions on manuscripts across broader areas of neuroscience. Increasingly, the editors at *JNeurosci* are using a consultation process adapted from that used by *eNeuro* to reach consensus between reviewers with differing recommendations, or to consult among editors about manuscripts that are not sent out for formal review. The goal of these consultation processes is to provide a more rapid decision to the authors, as well as a more focused idea of what would be needed for a revised manuscript to be appropriate for publication.

## Editorial Board and Content

At both journals, all editors are respected, working neuroscientists who publish articles in areas related to those they handle, and who, therefore, have firsthand knowledge publishing articles in neuroscience journals today. In terms of content, *JNeurosci* focuses on mechanistic studies that provide in-depth understanding of novel findings in neuroscience. *JNeurosci* has no upper or lower limit for the number of figures, but the reviewers and editors are asked to identify the most significant findings of the manuscript and to evaluate how the findings will change thinking in the neuroscience field. *eNeuro* has no constraints on the length of the paper or the number of figures, and is interested in publishing novel, but not necessarily mechanistic, findings that are of potential broad interest. As part of the process of differentiating the role of the two journals, the Brief Communication format, which focuses on important new observations that do not yet have a mechanistic underpinning, has been moved from *JNeurosci* to *eNeuro*. Along with important novel observations in neuroscience, *eNeuro* encourages the submission of papers on new methods, commentaries, negative results, important replication, and failure to replicate studies. Importantly, *eNeuro* does not accept papers that would require >3 months of work to be appropriate for publication, so if many more experiments are required, the paper might be recommended for re-review as a more in depth study at either journal.

## Summary

Although *JNeurosci* and *eNeuro* publish studies across the breadth of the neuroscience field and are focused on technical excellence, the format, review process, and types of articles published already differ substantially between the two journals. We anticipate that *JNeurosci* and *eNeuro* will continue to evolve, both by strategic decisions based on ongoing discussion between the editorial boards of the journals, and as a result of the natural evolution that occurs as authors choose where to submit their work. Both journals welcome input from the neuroscience community on how to provide options for publishing rigorous and insightful research, and on how these two Society for Neuroscience journals can best serve the field.

